# Effect of Nicotinamide on the Photolysis of Riboflavin in Aqueous Solution

**DOI:** 10.3797/scipharm.1507-04

**Published:** 2015-08-16

**Authors:** Iqbal Ahmad, Sofia Ahmed, Muhammad Ali Sheraz, Zubair Anwar, Kiran Qadeer, Adnan Noor, Maxim P. Evstigneev

**Affiliations:** 1 Baqai Institute of Pharmaceutical Sciences, Baqai Medical University, Toll Plaza, Super Highway, Gadap Road, Karachi 74600, Pakistan; 2 Department of Pharmaceutical Chemistry, Faculty of Pharmacy, University of Karachi, Karachi 75270, Pakistan; 3 Department of Physics, Sevastopol State University, Universitetskaya str. 33, Sevastopol 299053, Russia; 4 Department of Biological and Chemical Sciences, Belgorod National Research University, Pobeda str. 85, Belgorod 308015, Russia

**Keywords:** Riboflavin, Nicotinamide, Photolysis, Kinetics, Rate-pH profile

## Abstract

The photolysis of riboflavin (RF) in aqueous solution in the presence of nicotinamide (NA) by visible light has been studied in the pH range 1.0–12.0 and the various photoproducts have been identified as known compounds. RF has been determined in degraded solutions by a specific multicomponent spectrometric method in the presence of its photoproducts and NA. The second-order rate constants (*k*_2_) for the bimolecular interaction of RF and NA range from 0.54 (pH 1.0) to 9.66 M^–1^ min^–1^ (pH 12.0). The log *k*_2_–pH profile for the photolysis reaction follows a sigmoid curve showing a gradual increase in the rate of pH due to a change in the ionization behavior of the molecule. The lower rate in the acid region is probably due to protonation of the molecule since the cationic form of RF is less susceptible to photolysis than the neutral form. Similarly, a slowing of the rate in the alkaline region is due to anion formation of the molecule. NA is involved as an electron acceptor during the sequence of reactions and thus enhances the rate of photolysis of RF. Absorption and fluorescence measurements did not provide evidence for the complex formation between the two compounds under the present conditions.

## Introduction

Aqueous solutions of riboflavin (RF) degrade upon exposure to light [1–3] to give a number of inactive compounds under aerobic and anaerobic conditions [4–7]. Several studies have been carried out to evaluate the effects of solvent, pH, buffer, and light intensity/ wavelengths on the photodegradation reactions of RF [5, 6, 8–13]. The solubility of RF in aqueous solution is enhanced by nicotinamide (NA) in the concentration range of 10^–2^ M and above, due apparently to a molecular association/complex formation between the two vitamins [14–18]. Flavins also form charge-transfer complexes with NA through oxidoreduction [19–22]. Biological nicotinamide-dependent oxidoreduction consists of reversible 2e^–^ oxidoreduction of substrates. RF and NA are both components of the vitamin B-complex and multivitamin preparations and there is a possibility of interaction between the two components upon exposure to light. It is, therefore, necessary to study the effect of NA on the photolysis of RF to understand the nature and extent of this interaction. Moreover, the study has to be carried out over a wide range of pH to determine the pharmaceutically-useful pH range for optimum stability of RF in vitamin preparations. Some studies, including the effect of NA on the photolysis of cyanocobalamin [23] and that of RF on the photolysis of folic acid [24] and cyanocobalamin [25], have been reported.

## Materials and Methods

Riboflavin (RF), lumiflavin (LF), lumichrome (LC), and nicotinamide (NA) were obtained from Sigma Chemical Co., St. Louis, MO. 7,8-Dimethyl-10-(formylmethyl)isoalloxazine (formylmethylflavin, FMF) and carboxymethylflavin (CMF) were prepared according to the methods of Fall and Petering [26] and Fukumachi and Sakurai [27], respectively. All reagents and solvents used were of the purest form available from BDH, Poole, Dorset/Merck & Co., Whitehouse Station, NJ. The following buffer systems were employed: KCl–HCl, pH 1.0–2.0; citric acid-Na_2_HPO_4_, pH 2.5–8.0; Na_2_B_4_O_7_–HCl, pH 8.5–9.0; Na_2_B_4_O_7_–NaOH, pH 9.5–10.5; Na_2_HPO_4_–NaOH, pH 11.0–12.0. The ionic strength was 0.005 M in each case.

### Precautions

In view of the photosensitivity of RF, the experimental work was carried out in a dark chamber under subdued light. RF solutions containing NA were protected from light before photolysis. Freshly prepared solutions were used for each experiment to avoid any chemical or photochemical effects.

### Photolysis of RF

A series of 5×10^–5^ M solutions of RF (100 ml) was prepared in Pyrex flasks at the appropriate pH and a sufficient amount of NA was added to each flask to produce five dilutions in the concentration range of 0.5–2.5×10^–4^ M. The solutions were placed in a water bath maintained at 25±1°C and irradiated with a Philips HPL-N 125 W high-pressure mercury vapor fluorescent lamp (emissions at 405 and 435 nm, the latter wavelength overlapping the 445 nm band of RF), fixed horizontally at a distance of 25 cm from the center of the flasks. The solutions were continuously stirred by bubbling a stream of air into the flasks.

### Spectral Measurements

The spectral measurements on all the solutions were performed on the Shimadzu UV-240 Recording Spectrometer (Japan) using quartz cells of 10-mm path length.

### Fluorescence Measurements

The fluorescence measurements were carried out at room temperature on a double-beam JASCO FP-550 Spectrofluorimeter (Tokyo, Japan) equipped with a xenon arc lamp and double-grating monochromators using 1-cm quartz cells. The fluorescence intensity scale was calibrated with 0.1 mM RF solution (pH 7.0, 0.005 M phosphate buffer) as the standard for the relative fluorescence intensity measurements on RF solutions. The excitation wavelength was set at 444 nm and the emission was measured at 530 nm [28].

### Light Intensity Measurements

The intensity of the Philips HPL-N 125 W high-pressure mercury vapor lamp was determined by potassium ferrioxalate actinometry [29] as 1.14±0.12×10^17^ quanta s^–1^.

### Thin-Layer Chromatography

Thin-layer chromatographic (TLC) analysis of RF and its photoproducts in photolyzed solutions was carried out on 250-µm cellulose plates (Whatman CC 41) using the solvent systems: (a) 1-butanol–acetic acid–water (40:10:50, v/v, organic phase); (b) 1-butanol–1-propanol–acetic acid–water (50:30:2:18, v/v) [30]. The detection of flavin spots was made by their characteristic fluorescence under UV (365 nm) excitation.

### Assay Method

The assay of RF and its major photoproducts (FMF, LC, and LF) in degraded solutions was carried out by a specific multicomponent spectrometric method developed by Ahmad and Rapson [31]. The method is based on the pre-adjustment of photolyzed solutions to pH 2.0 (KCl–HCl buffer), extraction of LC and LF with chloroform, chloroform residue dissolved in acetate buffer (pH 4.5), and a two-component assay at 445 and 356 nm. The aqueous phase (pH 2.0) containing RF and FMF and any minor product (e.g. CMF) was subjected to a two-component assay at 445 and 385 nm. The UV and visible absorption spectra of RF and photoproducts [31] and the spectral changes observed during the photolysis of riboflavin solutions [6] have been reported. The method was validated in the presence of NA to ensure its specificity under the experimental conditions employed in this work. The reproducibility of the method was confirmed by analyzing several synthetic mixtures of riboflavin and its photoproducts in the presence of the highest concentration of NA (2.5×10^–4^ M) used in this work.

## Results and Discussion

### Nature of Photoproducts

TLC was used to monitor the composition of the photoproducts of RF in degraded solutions using solvent systems (a) and (b). The products detected upon comparison of their R_f_ values and characteristic fluorescence with those of the authentic compounds in both the solvent systems were:

pH 1–6 (40–50% photolysis), FMF, LC (major), CMF (minor).

pH 7–12 (60–80% photolysis), FMF, LC, LF (major), CMF (minor).

Fluorescence of spots: RF, FMF, LF, CMF–yellow green; LC–sky blue.

**Fig. 1:**
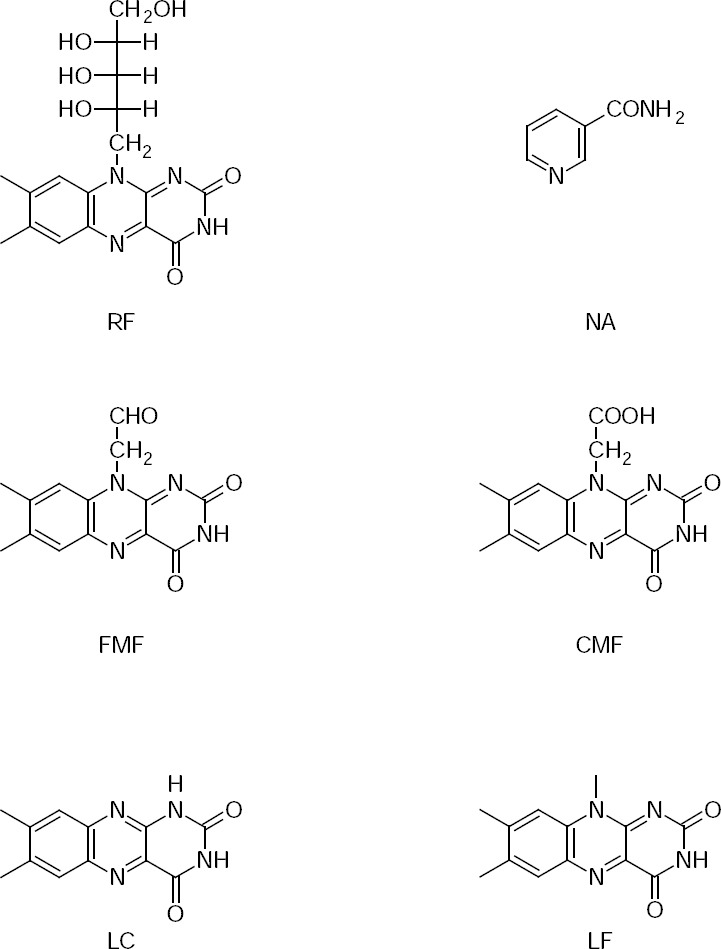
Chemical structures of RF, its photoproducts (FMF, LC, LF, CMF) and NA.

The major photoproducts of RF (FMF, LC, and LF) were formed faster in the presence of nicotinamide. FMF (a major intermediate in the photolysis of RF) [5] was rapidly degraded in the alkaline medium by hydrolysis to yield LC and LF [30]. CMF is an oxidation product of FMF and was formed in the concentration of less than 1%. All of these products have previously been identified [4–8,31], however, the rate of formation of these products would depend upon the reaction conditions. The chemical structures of RF, its photoproducts, and NA are shown in Fig. 1.

### Assay of RF and Photoproducts

The assay of RF and its major photoproducts (FMF, LC, LF) was carried out by a specific multicomponent spectrometric method [31] that has previously been employed for the study of RF photolysis [5, 6, 9, 10], FMF hydrolysis [30], and photolysis [32]. The method was validated in the presence of the highest concentration of nicotinamide (2.5×10^–4^ M) used in this study. The results of the analysis of varying concentrations of synthetic mixtures are given in Table 1. The reproducibility of the method lies within ±5%. This may be due to the complexity of the mixture and any interference from the minor products at the analytical wavelengths.

**Tab. 1:**
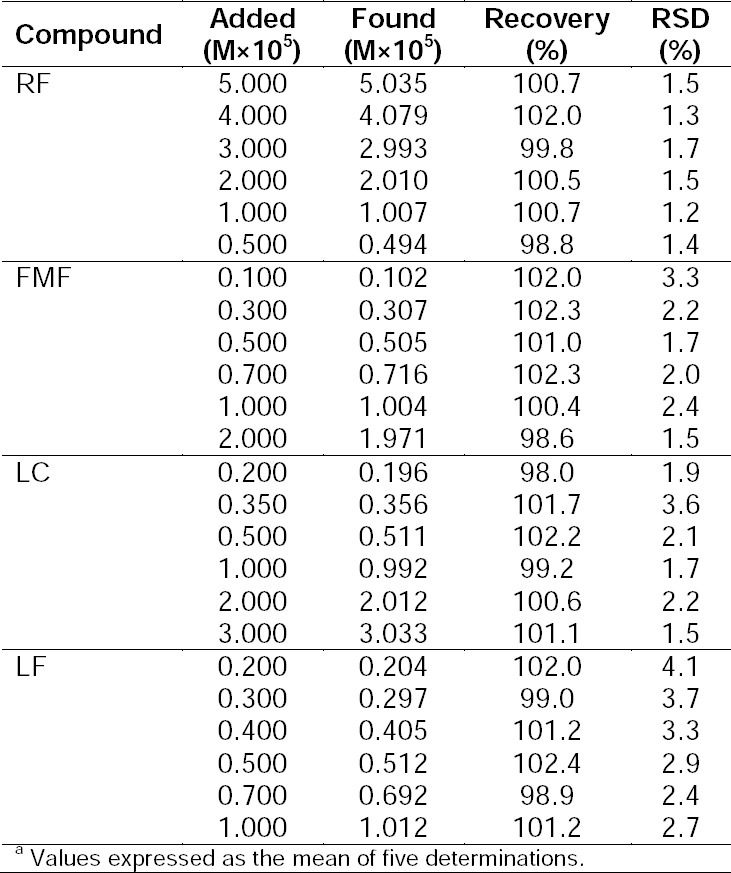
Analysis of synthetic mixtures of RF and photoproducts in the presence of NA (2.5×10^–4^ M)^a^.

### Kinetics of Photolysis

**Fig. 2:**
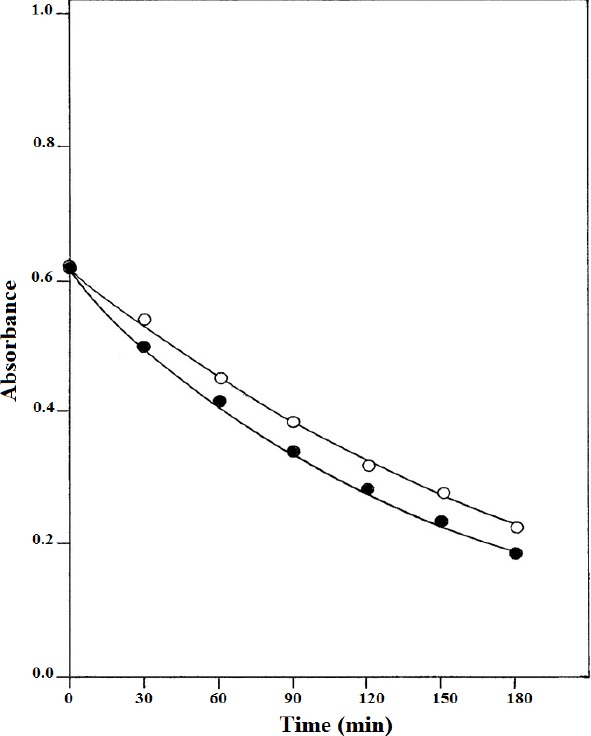
Plots of absorbance loss at 445 nm versus time for the photolysis of RF (5×10^–5^ M) in the presence (●) and absence (○) of NA (2.5×10^–4^ M).

In order to observe the influence of NA on the rate of photolysis of RF, absorbance measurements on the aqueous phase (after removal of the final degradation products, LC, and LF by chloroform extraction) were made at 445 nm and the values were plotted against time (Fig. 2). It is evident from the kinetic curves that the photolysis of RF is accelerated in the presence of NA. A complete analysis of the components of an RF solution photolyzed at pH 7.0 in the presence and absence of NA is given in Table 2. The composition of the photolyzed solutions of RF at 180 min of irradiation gave the following relative values of RF and the photoproducts:

**Tab. 2:**
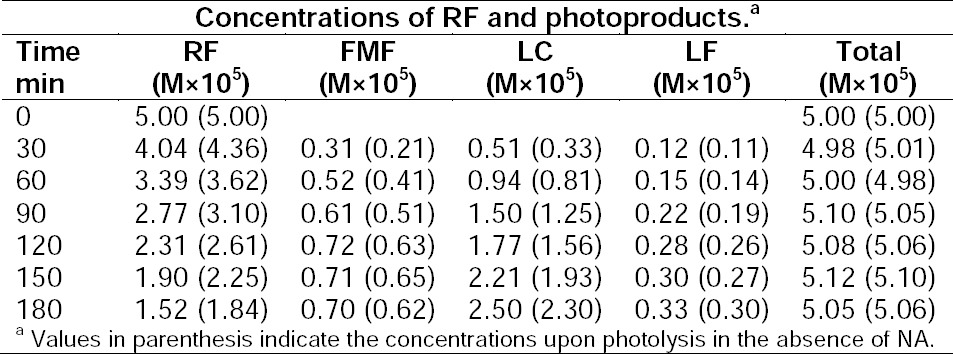
Photolysis of 5.0×10^–5^ M solution of RF in the presence of NA (2.5×10^–4^ M) at pH 7.0.

In the presence of NA: In the absence of NA:

RF 30.4%, FMF 14.0%, LC 50.1%, LF 6.6%. RF 36.8%, FMF 12.4%, LC 46.0%, LF 6.0%.

Thus, the photolysis of RF in the presence of NA (2.5×10^–4^ M) occurs to the extent of 70% compared to that in its absence being 63%, indicating that NA does influence the rate of photolysis of RF.

RF is known to undergo photolysis in aqueous solution by first-order kinetics [5,6]. Therefore, the log concentration values for the photolysis reactions carried out in the presence of NA at various pH values were plotted against time and the apparent first-order rate constants (*k*_obs_) were determined (Table 3). Further treatment of the kinetic data was carried out by plotting the values of *k*_obs_ against the respective molar concentrations of NA and the second-order rate constants (*k*_2_) for the interaction of RF and NA were determined from the slopes of the straight lines (Table 4). Since RF exists in several acid-base equilibria in the whole pH range, the rates of the reaction would depend on the susceptibility of particular species (cationic, zwitterionic, and anionic) to light activation and photodegradation.

**Tab. 3:**
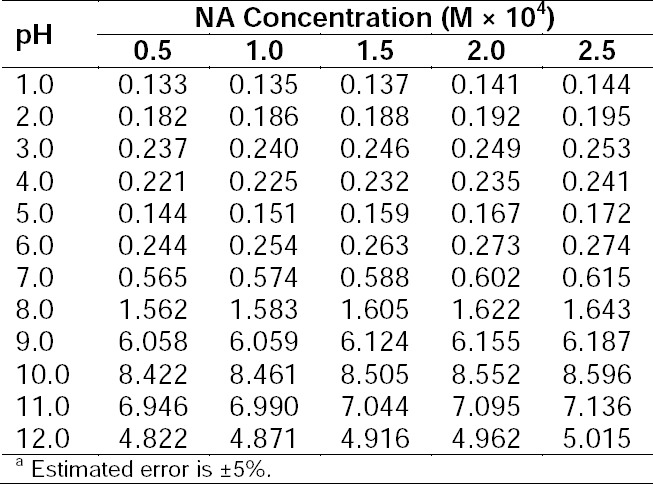
Apparent first-order rate constants (*k*_obs_×10^3^, min^–1^) for the photolysis of RF at pH 1.0–12.0 in the presence of NA (0.5–2.5×10^–4^ M)^a^.

**Tab. 4:**
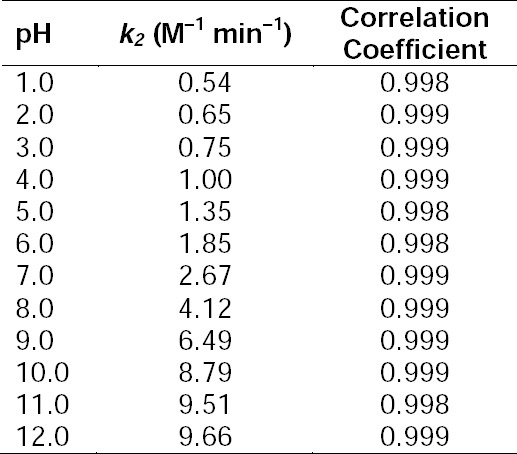
Second-order rate constants (*k*_2_, M^–1^ min^–1^) for the photochemical interaction of RF and NA at pH 1.0–12.0.

Irradiation of NA solutions (λ_max_ 261 nm) [33] with the lamp used in the study (visible emission) did not show any change in the absorption spectra of NA during the reactions.

### Rate–pH Profile

The rates of photodegradation reactions involving hydrolysis or oxidation may be influenced by pH due to acid-base catalysis or change in redox potentials. In pharmaceutical preparations, the rate–pH profiles were prepared to determine the pH of optimum stability for formulation purposes [34–38]. Liquid vitamin preparations are a complex system containing a number of vitamins which are liable to interaction and degradation upon exposure to light. Few reports have appeared on the study of the rate–pH profiles of binary systems including those on the effect of NA on the photolysis of cyanocobalamin [23] and the effect of RF on the photolysis of folic acid [24] and cyanocobalamin [25].

**Fig. 3:**
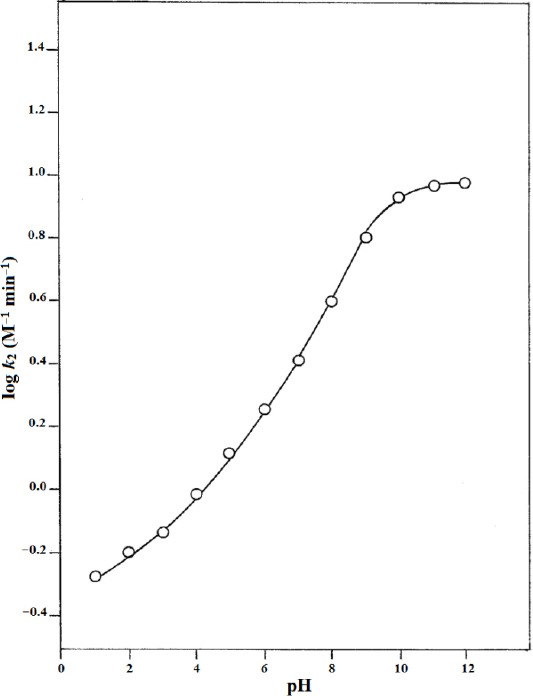
log *k*_2_–pH profile for the photolysis of RF in presence of NA.

In the present study, a log *k*_2_–pH profile has been constructed for the photochemical interaction of RF and NA in the pH range 1.0-12.0 and is shown in Fig. 3. The profile is represented by a sigmoid curve, indicating a gradual increase in the rate up to pH 10.0, followed by a leveling off in the pH range 10.0–12.0. RF (p*K*_a1_ 1.7, p*K*_a2_ 10.2) [39] and NA (p*K*_a_ 3.3) [33] both exist in the protonated form in the acid region and may be less susceptible to photo-activation and interaction in this state, resulting in a lower rate of photolysis in this region (Table 3). Both RF and NA are gradually deprotonated upon an increase in pH with a subsequent rise in the rate of the reaction. This may be due to greater susceptibility of the non-ionized forms of these compounds to photochemical interaction. The increase in rate with pH in the alkaline region may be due to increased reactivity of the excited triplet state [40]. The rate is considerably slowed down in the pH range 10.0–12.0 as a result of the ionization of RF (N–3) to form the anionic species. Such rate-pH profiles have been reported for the degradation of ascorbic acid [41], 5-fluorouracil [42], and phenobarbital [43].

A consideration of the photostability of RF as a function of pH shows that RF has the lowest rate of photolysis around pH 5 [5]. The log *k*_2_–pH profile (Fig. 3) also indicates that the rate of RF–NA interaction is low in the moderately acid range of pH 4–5. Therefore, this pH range appears to be suitable for the optimum photostability of RF in vitamin preparations. These preparations are normally formulated in the pH range 4–5.

### RF–NA Interaction

Several schemes have been proposed for the photoreduction of RF and related flavins to undergo degradation [9, 44–48]. In view of the effect of NA on the rate of photolysis of RF and the previous work, a scheme for the photolysis of RF in aqueous solutions in the presence of NA may be presented to express the sequence of reactions involved in the photoreduction of RF and its subsequent interaction with NA leading to an increase in the rate of the reaction (Scheme 1).

**Sch. 1:**
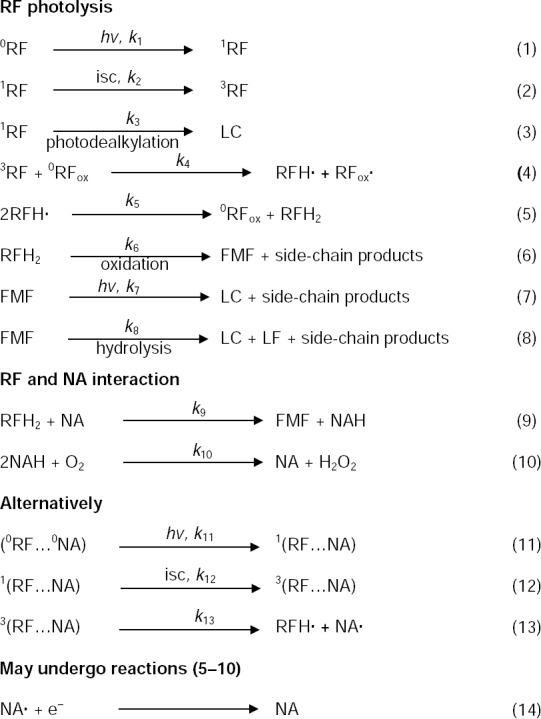
Scheme for the photolysis of RF in aqueous solution in the presence of NA.

The ground state RF molecule (^0^RF), upon absorption of light, is promoted to the excited singlet state (^1^RF) (1) which may be converted by intersystem crossing (isc) to the excited triplet state (^3^RF) (2) or may directly be converted to LC by photodealkylation (3). The ^3^RF may react with ^0^RF to form the semiquinone radicals (RFH·) (4). The disproportionation of the semiquinone radicals could lead to a reduced flavin (RFH_2_) (5) which is oxidized to FMF and side chain products (6). FMF is photolyzed to LC and side-chain products in acid medium (7) or hydrolyzed to LC and LF in neutral and alkaline media (8). Reactions (1)–(8) are well-established and their mechanisms have been elucidated by laser flash photolysis and other techniques [32, 45–49]. In the two additional steps, NA (an electron acceptor) [37] appears to be involved in the photolysis reaction. The kinetic data on the increase in the rate of photolysis of RF in the presence of NA can be explained on the basis of the interaction of the NA molecule with the photochemically-formed reduced flavin (RFH_2_) to undergo reduction to NAH (9) [19–21]. The reduced NA (NAH) is then oxidized to NA by O_2_ (10). Therefore, NA acts as an electron acceptor during the reaction, enhances the oxidation of RFH_2_ to FMF, and thus increases the rate of RF photolysis. Polarographic evidence for the interaction and redox reactions of flavins and NA has been obtained [19–21]. Alternatively, the ground state RF and NA may interact in the presence of light and be excited to the singlet state (11). The excited singlet state may then give rise to the excited triplet state (12) and form an exciplex [50] that may lead to a reduced semiquinone radical (RFH·) and an oxidized NA radical (NA· (13). RFH· may undergo the reactions (eq. (5)–(10)). The NA radical may accept an electron and be converted to NA (14).

The basic role of NA in RF solutions is to act as a solubilizing agent at high concentrations of RF [14–18], however, in the present study the concentration of RF (5×10^–5^ M) was low enough to be dissolved without the aid of NA. To examine the possibility of some form of association/complex formation between the two molecules, absorption and fluorescence measurements were carried out. Aqueous solutions containing RF and NA (pH 7.0, phosphate buffer), at the concentrations used in this work, did not show any change in the UV and visible absorption spectrum or in the fluorescence intensity compared to that of RF alone. This is in agreement with the observations of Coffman and Kildsig [16] to rule out the possibility of complex formation between the two molecules at a low concentration. However, any contribution from RF–NA complexation would be negligible under the present conditions.

In the light of the present study, the most probable role of NA in the photolysis of RF is to act as an electron acceptor as shown in the reactions (eq. (9)–(10)) so as to facilitate the formation of FMF and thus promote the photolysis reaction as indicated by the kinetic data. The reaction is influenced by the pH of the medium as a result of the ionization of RF and greater susceptibility of the RF triplet state to photolysis in alkaline medium [40].

## Conclusion

NA is known to interact with RF and enhance its solubility at a high concentration (~10^–2^M) in aqueous solutions. However, in dilute solutions of RF (5×10^–5^ M), there appears to be no interaction between RF and NA. An accurate spectrometric assay of RF shows that NA promotes the photolysis of RF at a low concentration probably by acting as an electron acceptor. The mutual interaction of the two compounds in the presence of light results in an enhanced degradation of RF. The kinetic data show that the degradation of RF at pH 7.0 is faster to the extent of 10% in the presence of NA. The rate–pH profile for the photochemical interaction of RF and NA represents a sigmoid curve, indicating an increase in the rate of reaction with pH. The cationic and anionic forms of riboflavin are less susceptible to photolysis both in the presence and absence of NA. The second-order rate constant for the interaction at pH 12.0 is about 20% faster compared to that of pH 1.0. A reaction scheme for the photolysis of RF in the presence of NA is presented.
